# Nonsupine Sleep Position Among US Infants

**DOI:** 10.1001/jamanetworkopen.2024.50277

**Published:** 2024-12-12

**Authors:** Guodong Ding, Anqi Peng, Yan Chen, Angela Vinturache, Yongjun Zhang

**Affiliations:** 1 Department of Pediatrics, Xinhua Hospital, Shanghai Jiao Tong University School of Medicine, Shanghai, China; 2 Clinical Medical College, Yangzhou University, Yangzhou, Jiangsu Province, China; 3 Department of Obstetrics & Gynecology, University of Alberta, Edmonton, Alberta, Canada

## Abstract

This cross-sectional study investigates temporal changes in the prevalence of nonsupine sleep position among US infants from 4 to 12 months of age.

## Introduction

Approximately 3500 sleep-related deaths among infants are reported annually in the US, including those from sudden infant death syndrome (SIDS), undetermined causes, and accidental suffocation and strangulation in bed.^1^ Ninety percent of SIDS cases occur before infants reach 6 months of age, with highest incidence between 1 and 4 months, and less common after 8 months of age.^1^ Safe sleep recommendations to place infants in a supine position were associated with a sharp decline in SIDS and other sleep-related mortality in the late 1990s,^2,3^ followed by a less pronounced downward trend thereafter.^2,4^ One-fifth of US mothers are placing their infants in a nonsupine sleep position within 6 months after birth.^5^ This report analyzed temporal changes in the prevalence of nonsupine sleep position among US infants at 4, 6, 9, and 12 months of age.

## Methods

This study used the National Survey of Children’s Health (NSCH) data from 2016 to 2022 (released on April 24, 2024) on sleep position among US infants aged 0 to 12 months. The NSCH is a nationally representative survey for monitoring the health and well-being of noninstitutionalized children aged 0 to 17 years.^6^ The NSCH survey has been conducted annually since 2016, compared with previous 4-year intervals. Changes in survey design have enabled the annual release of national data and comprehensive state-level estimates every 2 to 3 years. Annual data were collected from parents or other primary caregivers through mail- or web-based questionnaires. Because we used publicly available, anonymized data, the institutional review board deemed this study exempt from review. This study followed the STROBE reporting guideline. Nonsupine sleep position was assessed by asking respondents, “In which position do you most often lay this baby down to sleep now?”, with possible answers of “on side,” “on back,” or “on stomach.” Respondents who selected on side or on stomach were classified as placing their infant in a nonsupine sleep position. Trend significance in the prevalence of nonsupine sleep positions among US infants at 4, 6, 9, and 12 months of age, overall and by sociodemographic subgroups, was assessed using a survey-weighted logistic regression model, with the survey year as a continuous variable. The confounders chosen were (1) self-reported race and ethnicity, (2) family income, (3) education level, (4) maternal age at delivery, (5) household smoking, and (6) infant gestation.^5^ All analyses were performed using R version 4.3.2 (R Project for Statistical Computing) from May to June 2024. Two-tailed *P* < .05 indicated significance.

## Results

Among 9779 participants, 383 individuals with missing sleep position data were excluded, leaving 9396 infants (1321 [23.5%] Hispanic, 487 [11.6%] non-Hispanic Black, 6307 [52.4%] non-Hispanic White, and 1281 [12.4%] other race and ethnicity [American Indian or Alaska Native, Asian, Native Hawaiian and Other Pacific Islander, some other race, and 2 or more races]) in the study. There were 2978, 4512, and 6953 infants in the study of sleep positions at 4, 6, and 9 months, respectively.

The overall prevalence of nonsupine sleep position was 12.0%, 12.9%, 19.2%, and 23.0% at 4, 6, 9, and 12 months, respectively, and varied sociodemographically, with a substantially higher prevalence among Black infants and infants from low-income, low-education, and smoking households (Tables 1 and 2). There were no significant temporal changes in the prevalence of nonsupine sleep position at 4, 6, 9, and 12 months, from 2016 to 2022. However, downward trends in nonsupine sleep position at 9 and 12 months were found among other racial and ethnic groups and higher education households. In contrast, an upward trend in nonsupine sleep position occurred at 4 months among Hispanic infants.

**Table 1.** Trends in Prevalence of Nonsupine Sleep Position Among US Infants at Ages 4 and 6 Months From the National Survey of Children’s Health, 2016 to 2022^a^

Nonsupine sleep positioning^b^	2016		2017		2018		2019		2020		2021		2022		Overall		Absolute (relative) difference, 2022 vs 2016^c^	*P* value for trend
	No.	% (95% CI)	No.	% (95% CI)	No.	% (95% CI)	No.	% (95% CI)	No.	% (95% CI)	No.	% (95% CI)	No.	% (95% CI)	No.	% (95% CI)		
**Age 4 mo**																		
Total^d^	523	10.1 (4.9-15.2)	247	11.2 (4.5-17.9)	307	17.3 (8.6-26.0)	270	15.4 (6.1-24.6)	429	7.1 (2.9-11.4)	632	9.6 (5.6-13.6)	570	13.1 (8.0-18.3)	2978	12.0 (9.5-14.5)	3.1 (30.4)	.83
Race and ethnicity^e^^,^^f^																		
Hispanic	63	5.3 (0.1-10.4)	20	7.6^g^ (0.1-24.9)^h^	46	6.0 (0.5-14.8)^h^	41	4.0^g^ (0.1-12.9)^h^	67	3.8^g^ (0.4-10.4)^h^	94	12.2 (3.5-20.9)	97	23.2 (9.2-37.1)	428	9.2 (5.5-12.9)	17.9 (339.4)	.048
Non-Hispanic Black	24	20.4 (4.7-37.4)^h^	10	36.2^g^ (6.7-65.2)^h^	20	46.9 (19.0-74.8)	15	27.6 (7.8-55.1)^h^	33	18.3 (0.7-35.8)	31	14.9 (3.6-29.8)^h^	22	21.6 (5.2-40.3)^h^	155	27.3 (16.4-38.3)	1.2 (5.9)	.46
Non-Hispanic White	375	10.5 (2.7-18.3)	180	11.5 (3.5-19.4)	203	15.6 (3.2-28.1)	172	11.1 (6.8-16.7) ^h^	279	6.7 (0.8-12.7)	405	7.0 (1.7-12.3)	367	7.1 (2.8-11.5)	1981	10.0 (6.6-13.4)	−3.3 (−31.9)	.06
Other	61	11.6 (4.7-22.2)^h^	37	1.6^g^ (0.0-9.5)^h^	38	10.7 (2.9-24.8)^h^	42	28.7 (3.0-54.4)	50	5.2^g^ (0.5-13.7) ^h^	102	10.3 (1.3-19.4)	84	7.9 (0.2-15.5)	414	11.8 (5.2-18.4)	−3.7 (−32.1)	.97
Family income, % federal poverty level^i^^,^^j^																		
<100	65	13.9 (10.5-17.6)^h^	30	27.0 (1.1-52.8)	36	25.8 (6.7-44.9)	37	31.8 (4.6-59.1)	58	9.5 (6.4-12.9)^h^	83	14.7 (5.1-24.3)	80	24.5 (12.0-37.0)	389	21.1 (13.6-28.6)	10.6 (75.9)	.76
100-199	87	6.9 (1.4-12.3)	28	2.9^g^ (0.7-6.0)^h^	61	17.3 (4.9-29.6)	43	18.5 (0.7-36.2)	66	10.3^g^ (7.3-13.5)^h^	104	11.4 (1.7-21.1)	75	12.5 (1.8-23.2)	464	11.6 (7.0-16.2)	5.6 (81.7)	.19
200-399	164	11.5 (4.5-18.6)	81	19.4 (4.7-34.1)	101	21.1 (17.8-24.5)^h^	97	4.7 (0.2-9.1)	148	9.4 (2.7-16.1)	190	11.7 (1.6-21.7)	162	16.0 (3.6-28.3)	943	13.0 (8.2-17.9)	4.4 (38.6)	.51
≥400	207	7.4 (6.0-9.0)^h^	108	3.8 (0.5-7.2)	109	6.9 (5.1-9.1)^h^	93	12.0 (9.4-15.0)^h^	157	1.9 ^g^ (1.1-2.9)^h^	255	4.3 (0.4-8.1)	253	6.7 (1.7-11.6)	1182	5.9 (3.2-8.6)	−0.8 (−10.5)	.82
Highest education of adult in household^k^^,^^l^																		
Less than college degree^m^	150	13.7 (3.2-24.2)	76	18.3 (3.4-33.2)	108	18.4 (7.1-29.7)	101	19.3 (3.8-34.9)	159	10.5 (2.3-18.7)	194	16.3 (7.8-24.8)	165	17.8 (9.3-26.3)	953	16.4 (12.0-20.9)	4.1 (30.2)	.88
College graduate or higher	360	7.7 (2.8-12.5)	171	6.4 (1.2-11.7)	199	16.0 (2.5-29.5)	169	11.4 (1.8-20.9)	270	4.4 (0.7-8.2)	438	4.1 (1.3-6.9)	405	10.3 (3.7-16.8)	2012	8.4 (5.6-11.2)	2.6 (34.1)	.71
Maternal age at delivery^k^^,^^n^																		
≤30 y	262	10.7 (4.1-17.2)	121	16.7 (3.1-30.4)	154	16.1 (6.5-25.7)	128	22.0 (7.1-36.9)	193	5.7 (2.5-9.3)^h^	261	11.7 (4.3-19.1)	203	13.9 (5.7-22.0)	1322	14.1 (10.1-18.2)	3.2 (30.1)	.98
>30 y	236	9.8 (1.0-18.7)	125	6.7 (2.2-11.3)	150	18.3 (2.2-34.4)	139	7.4 (3.5-12.8)^h^	232	6.9 (2.2-11.5)	363	7.1 (2.8-11.4)	362	12.1 (5.4-18.7)	1607	9.5 (6.4-12.5)	2.2 (22.8)	.77
Smoker in household^k^^,^^o^^,^^p^																		
Yes	57	11.2 (4.0-21.5)^h^	24	32.7 (12.6-51.1)^h^	28	7.1 (0.1-18.3)^h^	22	42.2^g^ (20.7-63.6)^h^	50	13.1^g^ (4.5-24.3)^h^	51	23.1 (11.3-35.3)^h^	32	1.8^g^ (0.0-10.9)^h^	264	17.9 (6.5-29.3)	−9.5 (−84.3)	.69
No	460	10.2 (4.5-15.8)	222	8.9 (3.0-14.8)	272	16.2 (6.9-25.4)	245	13.0 (5.0-21.0)	370	6.4 (2.7-10.1)	566	7.6 (4.2-11.0)	524	13.2 (7.7-18.6)	2659	10.7 (8.4-13.1)	3.0 (29.6)	.81
Infant gestation, wk^k^^,^^q^																		
Preterm (<37)	50	19.4 (8.6-31.4)^h^	19	25.6^g^ (6.1-45.6)^h^	28	28.0 (10.7-44.9)^h^	26	2.8^g^ (0.0-13.2)^h^	50	4.7^g^ (0.5-13.7)^h^	59	10.3 (3.8-20.8)^h^	55	14.1 (1.5-26.8)	287	13.4 (5.6-21.1)	−5.3 (−27.4)	.22
Full term (≥37)	468	8.6 (4.5-12.8)	227	10.1 (3.8-16.3)	273	14.6 (5.6-23.6)	242	17.5 (7.0-28.0)	379	7.5 (2.7-12.4)	570	9.6 (5.3-13.9)	512	12.8 (7.2-18.3)	2671	11.6 (8.9-14.2)	4.1 (47.6)	.80
**Age 6 mo**																		
Total^d^	813	15.0 (9.9-20.1)	360	10.5 (5.6-15.3)	469	15.8 (9.4-22.1)	411	15.3 (8.2-22.4)	638	8.9 (5.3-12.4)	930	11.1 (7.5-14.6)	891	14.0 (10.0-17.9)	4512	12.9 (11.0-14.9)	−1.1 (−7.1)	.62
Race and ethnicity^e^^,^^f^																		
Hispanic	95	11.6 (4.2-19.0)	37	6.2 (0.7-18.2)^h^	62	5.0 (1.0-13.5)^h^	58	11.7 (0.8-22.6)	105	4.0 (1.0-9.5)^h^	132	15.0 (6.0-24.0)	149	23.3 (12.0-34.6)	638	11.0 (7.7-14.4)	11.7 (100.5)	.07
Non-Hispanic Black	34	20.1 (2.8-37.5)	18	32.9 (1.8-64.1)	33	49.8 (26.4-73.2)	21	28.3 (1.6-55.1)	42	19.7 (3.3-36.1)	50	13.8 (2.2-25.5)	33	20.2 (3.1-37.3)	231	27.2 (18.3-36.1)	0.1 (0.3)	.20
Non-Hispanic White	592	13.7 (6.8-20.6)	262	11.0 (5.1-16.8)	316	13.5 (5.0-21.9)	275	9.5 (0.9-18.0)	416	8.5 (3.7-13.4)	602	8.9 (4.5-13.2)	575	9.4 (5.7-13.2)	3038	10.7 (8.2-13.2)	−4.2 (−30.9)	.17
Other	92	24.7 (6.1-43.3)	43	1.3^g^ (0.0-8.2)^h^	58	10.7 (1.3-20.1)	57	30.7 (8.3-53.2)	75	13.3 (0.6-26.1)	146	9.8 (3.2-16.4)	134	9.8 (2.9-16.7)	605	14.6 (9.0-20.2)	−14.9 (−60.2)	.58
Family income, % federal poverty level^i^^,^^j^																		
<100	100	20.1 (6.1-34.2)	40	15.7 (10.9-20.5)^h^	57	26.9 (9.5-44.3)	51	31.1 (7.8-54.4)	79	14.9 (4.2-25.7)	126	20.9 (10.3-31.5)	119	23.3 (13.2-33.4)	572	22.1 (15.9-28.3)	3.2 (15.7)	.99
100-199	142	17.2 (7.2-27.1)	52	7.0 (0.8-13.3)	84	15.9 (5.5-26.4)	73	22.5 (8.6-36.3)	100	11.3 (0.2-22.4)	148	10.8 (3.2-18.4)	115	15.7 (6.8-24.7)	714	14.2 (10.4-18.1)	−1.4 (−8.2)	.82
200-399	252	12.2 (6.4-18.1)	113	16.3 (5.0-27.6)	154	13.8 (0.1-27.5)	138	6.9 (2.3-11.4)	219	11.8 (5.7-17.9)	281	10.2 (3.4-17.0)	267	16.2 (7.2-25.1)	1424	12.4 (8.9-15.8)	3.9 (32.1)	.73
≥400	319	10.9 (4.6-17.3)	155	6.1 (2.4-9.8)	174	11.0 (4.3-17.8)	149	9.0 (7.1-11.0)^h^	240	2.5 (0.3-4.7)	375	6.6 (2.5-10.7)	390	7.9 (3.9-11.9)	1802	7.5 (5.4-9.7)	−3.0 (−27.5)	.28
Highest education of adult in household^k^^,^^l^																		
Less than college degree^m^	245	20.7 (10.6-30.7)	113	13.5 (3.9-23.2)	161	16.9 (7.9-26.0)	153	22.6 (9.8-35.5)	227	12.9 (6.0-19.9)	285	17.5 (10.1-25.0)	252	21.0 (13.5-28.4)	1436	17.8 (14.2-21.5)	0.3 (1.6)	.93
College graduate or higher	548	11.5 (6.5-16.5)	247	8.1 (3.6-12.7)	308	14.7 (5.6-23.8)	258	9.2 (2.4-15.9)	411	5.8 (2.6-9.0)	645	6.3 (3.5-9.2)	639	10.2 (5.6-14.9)	3056	9.3 (7.3-11.4)	−1.3 (−11.1)	.28
Maternal age at delivery^k^^,^^n^																		
≤30 y	391	13.1 (7.5-18.7)	176	15.2 (5.3-25.1)	231	15.5 (7.9-23.1)	195	22.5 (10.6-34.4)	289	9.5 (3.6-15.4)	394	13.5 (7.1-19.8)	338	15.5 (9.2-21.8)	2014	15.1 (12.0-18.2)	2.4 (18.3)	.65
>30 y	385	18.3 (9.4-27.3)	182	6.8 (3.0-10.6)	235	15.6 (4.8-26.4)	211	7.9 (1.2-14.5)	345	7.4 (3.6-11.2)	524	8.6 (4.7-12.5)	543	11.9 (6.8-16.9)	2425	10.7 (8.2-13.2)	−6.5 (−35.3)	.25
Smoker in household^k^^,^^o^^,^^p^																		
Yes	95	24.9 (5.8-43.9)	36	31.5 (8.4-54.5)	43	12.3 (3.9-25.1)^h^	34	35.5 (19.7-53.5)^h^	71	14.6 (7.0-24.4)^h^	70	25.0 (3.9-46.1)	53	13.5 (5.5-25.3)^h^	402	22.2 (13.2-31.1)	−11.4 (−45.9)	.54
No	707	13.6 (8.7-18.4)	323	7.9 (3.7-12.1)	416	14.3 (7.7-20.9)	373	13.6 (7.3-19.9)	556	8.2 (4.8-11.5)	836	9.4 (6.1-12.6)	817	13.7 (9.5-17.8)	4028	11.5 (9.7-13.3)	0.1 (0.8)	.82
Infant gestation, wk^k^^,^^q^																		
Preterm (<37)	75	16.6 (8.6-26.3)^h^	29	21.7 ^g^ (8.0-39.7)^h^	39	22.4 (9.3-36.5)^h^	37	3.8 ^g^ (0.1-14.2)^h^	69	7.4 (2.4-16.1)^h^	89	9.8 (1.8-17.8)	93	12.0 (2.6-21.4)	431	12.1 (6.2-18.1)	−4.6 (−27.6)	.40
Full term (≥37)	730	15.0 (9.9-20.1)	328	9.7 (5.0-14.4)	424	14.1 (7.6-20.6)	372	17.0 (9.1-24.9)	566	9.1 (5.2-13.0)	836	11.2 (7.4-15.1)	793	14.1 (9.8-18.4)	4049	12.9 (10.8-14.9)	−0.8 (−5.6)	.84

^a^ All estimates, except sample sizes, are weighted.

^b^ Nonsupine sleep positioning was defined as sleep position where the baby predominantly lies on his or her side or stomach.

^c^ Absolute differences in estimated prevalence between 2016 and 2022 were calculated by treating each survey cycle as an indicator category, with the 2016 cycle as the reference. Values may not equal the difference between the beginning and ending estimates because of rounding. Relative difference, presented as a percentage, is the absolute difference divided by the prevalence in the referent category (2016) multiplied by 100.

^d^ *P *value for trend was adjusted for race and ethnicity, family income, highest education of adult in household, maternal age at delivery, smoker in household, and infant gestation.

^e^ Race and ethnicity (child’s Hispanic origin had 0.3% and race had 1.6% missing cases) was imputed by the Census Bureau using hot-deck imputation; other includes Asian, American Indian or Alaska Native, Native Hawaiian and Other Pacific Islander, some other race, and 2 or more races.

^f^ *P *value for trend was adjusted for family income, highest education of adult in household, maternal age at delivery, smoker in household, and infant gestation.

^g^ Number of individuals with nonsupine sleep positioning was less than 5.

^h^ 95% CIs were derived according to binomial distribution for standardization; all negative values have been replaced with 0.

^i^ Family income (16.6%) was imputed by the Census Bureau using sequential regression imputation methods.

^j^ *P *value for trend was adjusted for race and ethnicity, highest education of adult in household, maternal age at delivery, smoker in household, and infant gestation.

^k^ Numbers vary slightly in the subgroups due to missing data.

^l^ *P *value for trend was adjusted for race and ethnicity, family income, maternal age at delivery, smoker in household, and infant gestation.

^m^ Includes less than high school, high school, and some college or technical school.

^n^ *P *value for trend was adjusted for race and ethnicity, family income, highest education of adult in household, smoker in household, and infant gestation.

^o^ Household smoking exposure was determined based on responses to the query: “Is there any individual residing in your household who consumes cigarettes, cigars, or pipe tobacco?”

^p^ *P *value for trend was adjusted for race and ethnicity, family income, highest education of adult in household, maternal age at delivery, and infant gestation.

^q^ *P *value for trend was adjusted for race and ethnicity, family income, highest education of adult in household, maternal age at delivery, and smoker in household.

**Table 2.** Trends in Prevalence of Nonsupine Sleep Position Among US Infants at Ages 9 and 12 Months From the National Survey of Children’s Health, 2016 to 2022^a^

Nonsupine sleep positioning^b^	2016		2017		2018		2019		2020		2021		2022		Overall		Absolute (relative) difference, 2022 vs 2016^c^	*P* value for trend
	No.	% (95% CI)	No.	% (95% CI)	No.	% (95% CI)	No.	% (95% CI)	No.	% (95% CI)	No.	% (95% CI)	No.	% (95% CI)	No.	% (95% CI)		
**Age 9 mo**																		
Total^d^	1231	20.8 (16.0-25.7)	570	20.2 (13.1-27.4)	703	18.2 (12.8-23.6)	657	22.1 (15.1-29.2)	1015	18.0 (13.8-22.2)	1419	17.3 (12.9-21.8)	1358	17.9 (14.5-21.4)	6953	19.2 (17.2-21.3)	−2.9 (−13.8)	.36
Race and ethnicity^e^^,^^f^																		
Hispanic	143	23.9 (13.0-34.7)	61	16.6 (8.2-28.1)^g^	94	8.4 (0.6-16.2)	94	10.6 (3.0-18.1)	163	11.4 (3.4-19.4)	203	23.4 (11.7-35.0)	240	27.0 (18.1-35.9)	998	17.6 (13.1-22.1)	3.2 (13.3)	.57
Non-Hispanic Black	53	24.9 (5.3-44.4)	31	49.7 (21.4-78.0)	51	51.5 (33.3-69.7)	41	41.1 (20.0-62.3)	70	40.0 (22.2-57.7)	74	25.3 (7.9-42.6)	54	29.5 (13.1-45.9)	374	38.6 (30.4-46.8)	4.6 (18.7)	.59
Non-Hispanic White	886	14.2 (9.0-19.5)	401	16.2 (10.6-21.7)	475	14.1 (7.6-20.7)	428	18.1 (7.8-28.4)	639	15.5 (10.7-20.2)	937	13.8 (8.6-19.0)	863	11.9 (8.6-15.2)	4629	14.9 (12.5-17.3)	−2.4 (−16.6)	.47
Other	149	38.6 (22.6-54.7)	77	19.9 (2.3-37.5)	83	16.3 (4.3-28.4)	94	31.8 (13.6-49.9)	143	20.1 (8.4-31.8)	205	14.6 (7.2-21.9)	201	15.3 (8.4-22.2)	952	22.4 (16.9-27.8)	−23.3 (−60.4)	.04
Family income, % federal poverty level^h^^,^^i^																		
<100	152	25.9 (14.2-37.5)	65	30.5 (7.7-53.2)	89	26.4 (11.8-41.0)	93	34.8 (17.7-52.0)	141	22.6 (12.9-32.2)	179	25.6 (12.7-38.4)	184	27.6 (18.6-36.7)	903	27.6 (21.8-33.5)	1.8 (6.8)	.64
100-199	207	22.3 (11.7-32.8)	87	17.1 (4.2-29.9)	136	17.9 (9.0-26.8)	114	25.7 (12.9-38.5)	160	21.0 (11.1-30.9)	231	21.0 (10.9-31.1)	202	21.4 (13.3-29.5)	1137	20.8 (16.7-24.9)	−0.9 (−3.9)	.75
200-399	376	21.4 (13.2-29.5)	187	19.3 (10.8-27.7)	220	16.5 (5.1-28.0)	216	15.7 (6.8-24.6)	332	17.3 (10.6-24.1)	430	20.3 (10.3-30.2)	387	17.1 (10.1-24.1)	2148	18.1 (14.8-21.4)	−4.3 (−20.0)	.46
≥400	496	14.7 (8.9-20.6)	231	16.4 (8.1-24.8)	258	13.1 (6.9-19.3)	234	18.2 (8.7-27.6)	382	14.9 (7.8-22.0)	579	8.6 (5.2-12.0)	585	13.2 (9.1-17.3)	2765	14.1 (11.6-16.6)	−1.5 (−10.3)	.21
Highest education of adult in household^j^^,^^k^																		
Less than college degree^l^	366	24.2 (15.6-32.8)	182	18.4 (7.6-29.3)	254	19.9 (11.7-28.0)	243	26.2 (13.5-38.9)	367	19.9 (13.4-26.4)	436	26.2 (17.1-35.3)	398	25.3 (18.7-31.9)	2246	22.6 (19.0-26.2)	1.1 (4.6)	.65
College graduate or higher	840	18.8 (12.9-24.6)	388	21.5 (12.1-31.0)	449	16.4 (9.2-23.6)	414	18.7 (11.7-25.8)	648	16.5 (11.1-22.0)	983	10.5 (7.6-13.4)	960	14.1 (10.2-18.0)	4682	16.7 (14.3-19.1)	−4.7 (−25.1)	.03
Maternal age at delivery^j^^,^^m^																		
≤30 y	589	18.3 (12.1-24.5)	274	22.8 (11.0-34.5)	342	15.9 (9.2-22.6)	307	22.4 (13.1-31.7)	459	15.6 (10.1-21.1)	588	17.7 (11.2-24.2)	526	20.6 (15.0-26.3)	3085	19.0 (16.0-21.9)	2.3 (12.7)	.98
>30 y	582	22.9 (15.2-30.7)	289	18.6 (9.6-27.6)	354	20.3 (11.8-28.9)	341	17.4 (10.3-24.4)	550	19.3 (13.4-25.3)	816	16.7 (10.6-22.9)	818	15.5 (11.3-19.7)	3750	18.6 (15.9-21.3)	−7.4 (−32.4)	.26
Smoker in household^j^^,^^n^^,^^o^																		
Yes	141	22.9 (8.8-37.1)	63	26.9 (9.8-44.0)	73	21.7 (5.3-38.0)	60	52.5 (22.2-82.8)	108	19.0 (5.5-32.4)	114	29.6 (8.8-50.4)	83	19.7 (7.2-32.3)	642	27.4 (18.9-35.8)	−3.2 (−14.0)	.91
No	1072	20.5 (15.3-25.7)	503	19.6 (11.9-27.3)	615	16.5 (10.8-22.1)	589	18.6 (13.0-24.2)	890	18.0 (13.6-22.5)	1269	15.1 (10.8-19.4)	1240	17.5 (13.9-21.1)	6178	18.0 (15.9-20.0)	−3.0 (−14.8)	.34
Infant gestation, wks^j^^,^^p^																		
Preterm (<37)	116	20.0 (2.3-37.8)	50	20.7 (1.2-40.1)	65	19.4 (2.4-36.5)	69	10.1 (1.0-19.1)	112	17.7 (6.3-29.1)	144	23.6 (8.0-39.1)	143	11.5 (4.2-18.8)	699	17.5 (12.0-23.0)	−8.5 (−42.5)	.65
Full term (≥37)	1104	21.1 (16.1-26.1)	516	20.4 (12.7-28.1)	632	17.3 (11.7-23.0)	583	23.8 (16.0-31.5)	899	18.1 (13.6-22.5)	1270	16.6 (12.0-21.2)	1209	18.9 (15.1-22.6)	6213	19.4 (17.2-21.6)	−2.2 (−10.4)	.43
**Age 12 mo**																		
Total^d^	1754	24.7 (20.0-29.3)	759	23.9 (17.8-30.0)	940	23.7 (18.9-28.6)	881	21.9 (16.3-27.5)	1364	22.8 (18.7-27.0)	1839	21.2 (17.3-25.1)	1859	22.2 (18.7-25.8)	9396	23.0 (21.2-24.8)	−2.5 (−9.9)	.34
Race and ethnicity^e^^,^^f^																		
Hispanic	188	26.6 (14.4-38.9)	96	23.3 (7.5-39.1)	123	6.6 (0.8-12.4)	130	13.2 (5.6-20.9)	206	19.1 (10.0-28.2)	252	24.9 (14.7-35.1)	326	32.7 (23.8-41.6)	1321	21.3 (17.1-25.5)	6.0 (22.6)	.37
Non-Hispanic Black	78	27.9 (11.7-44.1)	37	50.8 (25.9-75.7)	66	59.6 (44.5-74.7)	54	39.0 (20.9-57.2)	86	44.4 (27.8-61.0)	89	31.9 (16.0-47.9)	77	34.1 (16.6-51.5)	487	41.9 (34.8-49.0)	6.2 (22.0)	.90
Non-Hispanic White	1268	18.4 (13.7-23.1)	520	19.1 (14.0-24.3)	632	20.8 (15.1-26.5)	581	19.5 (11.4-27.5)	878	18.7 (14.5-23.0)	1241	17.7 (13.3-22.2)	1187	15.3 (12.3-18.3)	6307	18.6 (16.6-20.6)	−3.1 (−16.8)	.29
Other	220	43.4 (30.2-56.6)	106	25.2 (9.7-40.7)	119	26.8 (13.4-40.1)	116	29.7 (13.7-45.6)	194	23.6 (13.1-34.0)	257	21.2 (12.5-30.0)	269	19.2 (12.7-25.7)	1281	27.0 (22.1-31.8)	−24.2 (−55.8)	.005
Family income, % federal poverty level^h^^,^^i^																		
<100	224	28.3 (17.1-39.4)	85	33.0 (12.9-53.1)	123	29.4 (16.8-42.0)	127	27.4 (14.4-40.5)	187	33.1 (20.8-45.5)	232	28.3 (17.0-39.6)	255	31.3 (21.1-41.5)	1,233	29.9 (24.8-35.0)	3.0 (10.6)	.95
100-199	287	24.8 (14.9-34.7)	111	19.9 (8.1-31.7)	182	21.0 (13.1-28.9)	149	25.8 (15.3-36.4)	211	22.0 (13.3-30.7)	290	24.5 (15.2-33.9)	279	24.2 (16.8-31.5)	1509	23.1 (19.5-26.7)	−0.6 (−2.6)	.72
200-399	538	26.0 (19.0-32.9)	246	21.1 (13.7-28.5)	279	22.9 (12.7-33.1)	280	16.3 (8.8-23.9)	435	24.0 (17.3-30.7)	558	21.6 (13.7-29.5)	522	20.5 (13.8-27.2)	2858	21.7 (18.8-24.7)	−5.5 (−21.2)	.37
≥400	705	20.2 (14.5-25.8)	317	23.4 (15.6-31.3)	356	22.2 (15.6-28.8)	325	20.6 (12.7-28.5)	531	17.4 (11.5-23.3)	759	15.4 (11.3-19.6)	803	18.7 (14.6-22.8)	3796	19.6 (17.3-21.9)	−1.5 (−7.3)	.11
Highest education of adult in household^j^^,^^k^																		
Less than college degree^l^	535	28.0 (19.6-36.4)	236	19.4 (10.3-28.6)	348	23.1 (15.9-30.3)	328	23.3 (13.6-33.0)	481	26.5 (19.4-33.6)	557	27.2 (19.3-35.0)	563	30.5 (23.1-37.8)	3048	25.2 (22.0-28.3)	2.5 (8.8)	.40
College graduate or higher	1191	22.4 (17.3-27.4)	523	27.3 (19.5-35.2)	592	24.4 (18.0-30.9)	553	20.7 (14.7-26.7)	883	20.0 (15.3-24.7)	1282	16.8 (13.4-20.3)	1296	17.5 (14.1-21.0)	6320	21.3 (19.2-23.3)	−4.8 (−21.6)	.007
Maternal age at delivery^j^^,^^m^																		
≤30 y	832	23.7 (16.5-30.9)	369	27.9 (18.2-37.5)	451	20.8 (14.5-27.0)	416	22.3 (15.0-29.7)	625	20.0 (14.8-25.1)	757	20.9 (15.1-26.8)	726	24.0 (18.3-29.7)	4176	22.8 (20.1-25.4)	0.3 (1.1)	.44
>30 y	830	25.5 (18.9-32.0)	379	21.1 (13.3-28.8)	480	26.2 (18.9-33.6)	453	18.0 (12.2-23.9)	728	24.8 (18.7-30.9)	1059	20.9 (15.6-26.2)	1114	20.7 (16.1-25.2)	5043	22.5 (20.1-24.8)	−4.8 (−18.9)	.59
Smoker in household^j^^,^^n^^,^^o^																		
Yes	205	27.5 (15.2-39.8)	87	28.0 (13.5-42.4)	110	25.6 (11.2-40.0)	85	42.1 (16.7-67.5)	148	29.9 (16.1-43.8)	143	35.8 (18.7-52.9)	121	18.2 (8.2-28.2)	899	30.2 (23.5-36.9)	−9.3 (−33.7)	.97
No	1528	24.3 (19.2-29.4)	665	23.4 (16.9-30.0)	810	23.0 (17.8-28.2)	784	19.4 (14.7-24.1)	1191	21.7 (17.4-26.0)	1653	18.9 (15.1-22.8)	1693	22.4 (18.6-26.2)	8324	21.9 (20.1-23.8)	−1.9 (−7.8)	.29
Infant gestation, wks^j^^,^^p^																		
Preterm (<37)	164	20.9 (6.6-35.1)	76	33.6 (15.6-51.6)	90	24.5 (9.7-39.3)	85	11.3 (2.6-19.9)	146	19.5 (9.3-29.6)	179	23.2 (9.9-36.5)	196	14.4 (7.3-21.6)	936	20.9 (16.1-25.8)	−6.4 (−30.8)	.31
Full term (≥37)	1574	25.2 (20.2, 30.2)	677	23.1 (16.6-29.6)	841	23.6 (18.4-28.8)	790	23.2 (17.1-29.3)	1212	22.9 (18.5-27.4)	1654	21.0 (16.9-25.1)	1653	23.2 (19.3-27.1)	8401	23.2 (21.2-25.1)	−1.9 (−7.7)	.50

^a^ All estimates, except sample sizes, are weighted.

^b^ Nonsupine sleep positioning was defined as sleep position where the baby predominantly lies on his or her side or stomach.

^c^ Absolute differences in estimated prevalence between 2016 and 2022 were calculated by treating each survey cycle as an indicator category, with the 2016 cycle as the reference. Values may not equal the difference between the beginning and ending estimates because of rounding. Relative difference, presented as a percentage, is the absolute difference divided by the prevalence in the referent category (2016) multiplied by 100.

^d^ *P *value for trend was adjusted for race and ethnicity, family income, highest education of adult in household, maternal age at delivery, smoker in household, and infant gestation.

^e^ Race and ethnicity (child’s Hispanic origin had 0.2% and race had 1.8% missing cases) was imputed by the Census Bureau using hot-deck imputation; other includes Asian, American Indian or Alaska Native, Native Hawaiian and Other Pacific Islander, some other race, and 2 or more races.

^f^ *P *value for trend was adjusted for family income, highest education of adult in household, maternal age at delivery, smoker in household, and infant gestation.

^g^ 95% CIs were derived according to the binomial distribution for standardization; all negative values have been replaced with 0.

^h^ Family income (16.6%) was imputed by the Census Bureau using sequential regression imputation methods.

^i^ *P *value for trend was adjusted for race and ethnicity, highest education of adult in household, maternal age at delivery, smoker in household, and infant gestation.

^j^ Numbers vary slightly in the subgroups due to missing data.

^k^ *P *value for trend was adjusted for race and ethnicity, family income, maternal age at delivery, smoker in household, and infant gestation.

^l^ Includes less than high school, high school, and some college or technical school.

^m^ *P *value for trend was adjusted for race and ethnicity, family income, highest education of adult in household, smoker in household, and infant gestation.

^n^ Household smoking exposure was determined based on responses to the query: “Is there any individual residing in your household who consumes cigarettes, cigars, or pipe tobacco?”

^o^ *P *value for trend was adjusted for race and ethnicity, family income, highest education of adult in household, maternal age at delivery, and infant gestation.

^p^ *P *value for trend was adjusted for race and ethnicity, family income, highest education of adult in household, maternal age at delivery, and smoker in household.

## Discussion

To our knowledge, this study is the first to simultaneously investigate sleep positions among US infants in 4 different age groups. We found that the overall prevalence of nonsupine sleep positions during infancy remained stable over the 7-year study period, suggesting no changes in parental behavior toward the sleep hygiene of their infants. The prevalence was higher among infants from lower socioeconomic status households, consistent with similar sociodemographic patterns observed in previous studies.^5^ Evidence-based interventions that promote safe sleep practices, particularly among younger age groups where SIDS is more prevalent, could help reduce sleep-related infant mortality. We acknowledge that the predominance of the non-Hispanic White population may limit the generalizability of our findings to other populations.

## Notes

Supplement.
Data Sharing Statement
