# Supplementary material for: Nonsupine Sleep Position Among US Infants
Source: JAMA Netw Open. 2024 Dec 12;7(12):e2450277. doi: 10.1001/jamanetworkopen.2024.50277 (PMC11638792; doi:10.1001/jamanetworkopen.2024.50277)
Supplement: Supplement. — Data Sharing Statement [file jamanetwopen-e2450277-s001.pdf]

Data Sharing Statement

Ding. Nonsupine Sleep Position Among US Infants. *JAMA Netw Open*. Published December 12, 2024. doi:10.1001/jamanetworkopen.2024.50277

Data
Data available: Yes
Data types: Deidentified participant data
How to access data: Data sharing will be available from YZ upon a reasonable request. Electronic address: zhangyongjun@sjtu.edu.cn.
When available: beginning date: 12-01-2024, end date: 12-01-2025

Supporting Documents
Document types: Statistical/analytic code
How to access documents: Data sharing will be available from YZ upon a reasonable request. Electronic address: zhangyongjun@sjtu.edu.cn.
When available: beginning date: 12-01-2024, end date: 12-01-2025

Additional Information
Who can access the data: Data sharing will be available from YZ upon a reasonable request. Electronic address: zhangyongjun@sjtu.edu.cn.
Types of analyses: Data sharing will be available from YZ upon a reasonable request. Electronic address: zhangyongjun@sjtu.edu.cn.
Mechanisms of data availability: Data sharing will be available from YZ upon a reasonable request. Electronic address: zhangyongjun@sjtu.edu.cn.
Any additional restrictions: Data sharing will be available from YZ upon a reasonable request. Electronic address: zhangyongjun@sjtu.edu.cn.
